# Intrasession and Intersession Reproducibility of Artificial Scotoma pRF Mapping Results at Ultra-High Fields

**DOI:** 10.1523/ENEURO.0087-22.2022

**Published:** 2022-09-21

**Authors:** David Linhardt, Maximilian Pawloff, Michael Woletz, Allan Hummer, Martin Tik, Maria Vasileiadi, Markus Ritter, Garikoitz Lerma-Usabiaga, Ursula Schmidt-Erfurth, Christian Windischberger

**Affiliations:** 1 High Field MR Center, Center for Medical Physics and Biomedical Engineering, Medical University of Vienna, 1090 Vienna, Austria; 2 Department of Ophthalmology, Medical University of Vienna, 1090 Vienna, Austria; 3 BCBL, Basque Center on Cognition, Brain and Language, 20009 Donostia-San Sebastián, Gipuzkoa, Spain

**Keywords:** fMRI, high-resolution MRI, pRF mapping, retinotopy, scotoma

## Abstract

Functional magnetic resonance imaging (fMRI) combined with population receptive field (pRF) mapping allows for associating positions on the visual cortex to areas on the visual field. Apart from applications in healthy subjects, this method can also be used to examine dysfunctions in patients suffering from partial visual field losses. While such objective measurement of visual deficits (scotoma) is of great importance for, e.g., longitudinal studies addressing treatment effects, it requires a thorough assessment of accuracy and reproducibility of the results obtained. In this study, we quantified the reproducibility of pRF mapping results within and across sessions in case of central visual field loss in a group of 15 human subjects. We simulated scotoma by masking a central area of 2° radius from stimulation to establish ground-truth conditions. This study was performed on a 7T ultra-high field MRI scanner for increased sensitivity. We found excellent intrasession and intersession reproducibility for the pRF center position (Spearman correlation coefficients for *x*, *y*: >0.95; eccentricity: >0.87; polar angle: >0.98), but only modest reproducibility for pRF size (Spearman correlation coefficients around 0.4). We further examined the scotoma detection performance using an automated method based on a reference dataset acquired with full-field stimulation. For the 2° artificial scotoma, the group-averaged scotoma sizes were estimated at between 1.92° and 2.19° for different sessions. We conclude that pRF mapping of visual field losses yields robust, reproducible measures of retinal function and suggest the use of pRF mapping as an objective method for monitoring visual deficits during therapeutic interventions or disease progression.

## Significance Statement

Population receptive field (pRF) mapping using functional magnetic resonance imaging (fMRI) is perfectly suited for the investigation of the retinotopic organization on the visual cortex. Expanding its influence from neuroscience toward more clinical areas of applications in patients suffering from visual field loss requires the assessment of the accuracy and reproducibility of the method. Within this work, we demonstrate the robustness of the method within as well as between two scanning sessions. This will lay the foundation for any future pRF mapping research in patients suffering from retinal disease.

## Introduction

One of the most important characteristics of the visual system is that the organization of the retinal visual field is preserved throughout the visual pathway until the visual cortex. In other words, neighboring areas on the visual field correspond to neighboring areas on the visual cortex.

This retinotopic organization can be ideally studied by noninvasive brain mapping using functional magnetic resonance imaging (fMRI). This was first demonstrated almost three decades ago by Engel et al. (1994) using contracting or expanding rings and rotating wedges (Sereno et al., 1995). These periodically appearing stimulus patterns induce sinusoidal activation patterns in the visual cortex, where the phase shift is encoding for the respective eccentricity or polar angle respectively.

A more advanced approach for investigating retinotopic organization using fMRI is population receptive field (pRF) mapping, which was introduced by Dumoulin and Wandell (2008). Because of the retinotopic organization, neighboring neurons within one voxel have comparable retinotopic properties and can therefore be combined into pRFs. Following, every voxel on the visual cortex is typically described by one pRF which is modelled as a 2D Gaussian function on the visual field. pRF modeling can be extended to implement surround suppression (Zuiderbaan et al., 2012) as the difference of two Gaussian functions, which can improve modeling in early visual areas. Further studies showed nonlinearities when only parts of a pRF are stimulated simultaneously (Kay et al., 2013). Novel pRF models implemented by Aqil et al. (2021) combine both surround suppression and spatial nonlinearities, with the promise of increased model power in pRF analyses. An extension to elliptical Gaussians is also possible, however, studies have shown that early visual cortex pRFs are almost circular (Zeidman et al., 2018; Lerma-Usabiaga et al., 2021). Besides improvements to the pRF method, novel deep learning models predicting retinotopic parameters based on subject-specific anatomic properties (Ribeiro et al., 2021) could complement and improve pRF mapping in the future.

In pRF analysis, model time courses are defined by the sequence of stimulation patterns and the receptive field model whose parameter sets are optimized for every voxel independently. A major advantage of the pRF mapping approach is the extension of possible stimulus configurations to nonperiodic patterns as the classic moving bar stimulus (Dumoulin and Wandell, 2008) or simultaneous wedges and rings (Alvarez et al., 2015). Although overall results are largely comparable, the exact choice of the stimulation pattern can affect the pRF results in a systematic way (Linhardt et al., 2021). An additional effect on pRF mapping results can arise from the choice of analysis tools and the implementation of hemodynamic response function (HRF) fitting (Lerma-Usabiaga et al., 2020).

It has also been shown that the method of pRF mapping is well-suited for the investigation of patients suffering from partial visual field loss because of retinal disease (Silson et al., 2018; Ritter et al., 2019; Carvalho et al., 2021; Prabhakaran et al., 2021). Compared with microperimetry, the ophthalmologic gold standard for visual field examination, fMRI is advantageous as no active response from the patient is necessary. Still, pRF mapping in patients poses several challenges.

An elegant strategy to establish ground-truth conditions in pRF mapping is the use of artificial scotomata, where visual field losses are simulated by masking stimulations in well-defined areas. While several studies have shown that artificial scotomata can be detected using pRF mapping (Hummer et al., 2018; Prabhakaran et al., 2021); however, some estimation bias may occur, as shown by Binda et al. (2013), using extensive simulations.

Clearly, to use pRF mapping for scotoma assessment, it is necessary to determine the reproducibility of such scotoma estimation methods. For full-field stimulation, studies have already assessed pRF reproducibility. van Dijk et al. (2016) evaluated pRF reliability in a group of 16 healthy subjects using the simultaneous wedges and rings aperture, revealing natural images rather than a standard checkerboard pattern, which is designed for enhancing response in higher visual areas (Silson et al., 2015). This showed high reproducibility of the pRF center position, but lower reproducibility for the pRF size parameter, based on full-field stimulation.

While this is a promising starting point, scotoma reproducibility assessment requires examining scotomas. Therefore, in this work, we measured healthy subjects multiple times using a standard moving bar stimulus revealing a flickering checkerboard pattern with artificial scotoma to mimic visual field losses. On these artificial scotoma mapping results, we assess the intrasession and intersession reproducibility of pRF mapping results to provide a basis for further fMRI retinotopy studies in patients with retinal disease. In order to maximize sensitivity, all scans were performed on a 7T MRI.

## Materials and Methods

### Subjects

For the main study, 20 healthy participants (10 female, 10 male; age: 25.9 ± 3.6) were recruited and scanned with artificial scotoma stimulation (see below). An additional group of 10 healthy subjects (four female, 6 male; age: 25.0 ± 2.8) were scanned as a full-field stimulation reference group. Subjects of the study group were attested for good sight (refractive error <6 dioptres) and no significant ocular disease. Further, no subject had a history of trauma or eye surgery. Subjects were naive to the task and were only instructed right before the first session of the experiment. Subjects gave informed written consent and received financial compensation for their participation. The research project was approved by the local ethics committee.

### MRI measurements

All MRI measurements in this study were performed at a magnetic field strength of 7T using a 32-channel head coil on a Siemens MAGNETOM scanner (Siemens Healthineers). Every subject participated in two sessions, on average 17.9 ± 11.6 d apart. For the acquisition of functional data, the CMRR EPI sequence (Moeller et al., 2010) was used with a spatial resolution of 1 mm isotropic and the following parameters: TE = 25.2 ms, TR = 2000 ms, matrix size = 128 × 128, field-of-view 128 × 128 mm, echo-spacing = 1 ms, flip angle = 70°, GRAPPA acceleration = 2, slice spacing = 10%. High spatial resolution imaging requires long readout times that can introduce spatial blurring effects (Windischberger and Moser, 2000). Based on the acquisition parameters of this study, the resulting full-width-at-half-maximum is 1.06 mm, i.e., only a slight increase of 6%.

Every run lasted for 5 min 36 s resulting in 168 acquired volumes. Every volume contains 32 slices of the participant’s occipital pole region, aligned perpendicular to the calcarine sulcus to capture early visual cortex areas. To later correct functional images for geometric distortions using FSL topup (Andersson et al., 2003; Smith et al., 2004), two short EPI measurements with opposite phase-encoding directions were performed. Additionally, anatomic full-brain images were recorded using a magnetization-prepared rapid gradient-echo (MPRAGE) sequence (0.7 mm isotropic resolution, TE = 3.66 ms, TR = 1960 ms).

Subjects were able to see the stimulus through a mirror, mounted inside the head coil. To minimize reflections inside the scanner bore, the rear-projection screen was moved as close to the head-coil as possible without reducing visual field coverage. This resulted in an average distance between the subject’s eye and the screen of 62 cm. One of the participant’s eyes was covered using an eyepatch. This is necessary to ensure monocular vision matching the experimental setup as used in patient measurements, where the focus lies on pathologies in one eye only.

Stimulus aperture consisted of a bar, revealing an 8 Hz reversing checkerboard and moving through the central 14° visual field of view (Dumoulin and Wandell, 2008) on gray background. The bar had a width of 1.75° and jumped after every TR for 0.8° to cross the visual field in 18 steps.

For the study group subjects, patient-typical central visual field losses were mimicked by masking the central 4° diameter (2° radius) of the visual field to permanently show the gray background corresponding to the artificial scotoma. After every crossing, the bar and the underlying checkerboard were rotated clockwise by 45°. Each run comprised eight crossings. After every second (diagonal) crossing, a blank period of 12 s was shown as a baseline. Participants were instructed to fixate a central dot and report the pseudo-randomized color changes between two high-contrast colors for the assessment of patient attention and compliance. One study group subject also underwent one full-field stimulation session.

Subject exclusion criteria included movement (mean framewise displacement >1 mm; Power et al., 2012) and insufficient fixation performance/attention (<85% correctly reported fixation dot color changes). Whenever at least one of the recorded runs per subject exceeded these thresholds, all data of this subject were excluded from the analysis.

The reference group was measured for one session with the same experimental setup as described above but using full-field stimulation. These data are required to implement automatic scotoma size estimation as introduced by Hummer et al. (2018). Functional data of the visual cortex were acquired with 1 mm isotropic resolution and TR = 1000 ms, which results in the acquisition of 336 volumes per run. The used aperture (moving bar revealing a reversing checkerboard pattern) behaved as described above, however, the discrete step width was 0.4° for 36 steps because of the differing repetition time. Each subject was measured for two runs, which were averaged before pRF analysis.

### Analysis

Data analysis was performed using a custom preprocessing pipeline including the following steps: slice-time correction using SPM12 (https://www.fil.ion.ucl.ac.uk/spm/) in MATLAB 9.6 (MATLAB, 2018), realignment (SPM) as well as correction for geometric distortions using FSL topup. No spatial smoothing was applied to the functional data. The anatomic MPRAGE image was bias-field corrected and forwarded to the FreeSurfer (https://surfer.nmr.mgh.harvard.edu/) *recon-all* segmentation pipeline to obtain a white-matter mask. This automated segmentation was manually corrected for segmentation as well as topological errors and used as input for the pRF analysis.

Functional (pRF) analysis was performed using the MATLAB toolbox mrVista (https://github.com/vistalab/vistasoft) based on Dumoulin and Wandell (2008). Within this analysis, the sequence of stimulus patterns defines specific time courses for every position (*x*, *y*) in the visual field. Because of the retinotopic organization, these time courses, convolved with the HRF, can be found in specific regions of the visual cortex. The corresponding pRF mapping parameters are determined by finding the best-fitting model in each visual cortex voxel.

The standard single Gaussian approach was chosen as the primary model type for the time course creation within this work. For this modeling approach, the possible pRF models on the visual field are defined via a single pRF size parameter σ, in addition to the position parameters *x* and *y*. Two-γ HRFs were fitted separately for each run.

For comparison with the standard analysis approach described above, we also applied two models with different parameter spaces. The first approach is equivalent to the standard model but uses a canonical HRF, i.e., no individual, run-specific HRFs are fitted and all subjects and runs used the same HRF. The second applied model is based on the work of Kay et al. (2013) and additionally accounts for spatial nonlinearities. All model fittings are performed as implemented in the vistasoft toolbox, following a five-stage coarse-to-fine fitting procedure.

The primary visual cortex (V1) was manually delineated on cortex maps based on the borders between areas located at the site of phase reversals in the polar angle parameter of the first run. In total, six analyses were performed in each subject:

1. Session 1, run 1
2. Session 1, run 2
3. Session 2, run 1
4. Session 2, run 2
5. Session 1, run 1 + run 2 combined
6. Session 2, run 1 + run 2 combined

Combined datasets were obtained by averaging run 1 and run 2 of the respective session before the analysis. Visualization of the pRF results was performed as parameter maps overlaid to the surface of the V1 and in the visual field space as coverage maps (Amano et al., 2009). Those coverage maps include information of every voxel’s pRF center as gray dot as well as the maximum surface of the corresponding Gaussian receptive field functions in the color coding.

It is important to note that the fitting routine is naive to the artificial central scotoma, i.e., fitting is performed assuming full-field stimulation. This is done to mimic measurements on patients, where the exact extent of the visual field loss is unknown and cannot be accurately modelled a priori.

### Correlation

All runs within one session were realigned and, additionally, the runs of the second session were coregistered to the first session. All available datasets for one subject were thus located in the same space and the white-matter segmentation and mrVista alignment was applied to all datasets of a subject to perform direct, voxel-to-voxel comparisons within and between sessions. Data were restricted to voxels located in the V1. Only voxels where the model was able to explain >10% of the variance in the data (variance explained) were included in future analyses.

For the assessment of the reliability of the pRF parameters eccentricity, polar angle and pRF size, Spearman’s correlation coefficients were calculated. Correlations were calculated between the two runs of each session, between sessions for each run and between averaged session 1 and session 2 results.

As correlation coefficients are not normally distributed, single-subject results were Fisher z transformed (Fisher, 1915), averaged and transformed back to obtain the averaged correlation across the subject group.

In order to assess systematic differences in correlation values for pRF center positions in Cartesian (*x*, *y*) and polar (radius, angle) coordinates, a simulation was performed. For this, a linear grid of data points was used. pRF positions were shifted using Gaussian noise and the Spearman’s correlation coefficient was calculated in polar and Cartesian coordinates. The process was repeated 1000 times and resulting correlation coefficients were averaged. Simulations were also repeated without grid points within the central 2° radius to assess possible effects of foveal scotomata.

### Scotoma border detection

Based on the work of Hummer et al. (2018), we used kernel density estimation (KDE) to determine scotoma borders. Here, pRF center density is integrated over the polar angle, discarding pRF size information, and displayed as a smoothed histogram over eccentricity. With the position in the diagram x and the pRF’s distance from the center of the field of view $r_{vox}$, the kernel is defined as

$$KDE(x,r_{vox})=\text{exp}\left(-\frac{{\left(x-r_{vox}\right)}^{2}}{2w^{2}}\right).$$

The sum over all pRF centers kernels

$$KDE_{pRF}\left(x\right)=\frac{1}{n\sqrt{2\pi w^{2}}}\underset{vox}{\sum }KDE(x,r_{vox})$$

defines the final KDE curve, with the bandwidth w calculated, according to Scott’s rule (Scott, 1992):

$$w=3.5\sigma_{r}n^{-\frac{1}{3}},$$

the eccentricity parameter’s variance $\sigma_{r}$ and the number of data points n. This calculation is performed independently for every subject and then averaged across the group. Scotoma borders were estimated as described by Hummer et al. (2018). In short, we compare the pRF center density of a single-subject result including an artificial scotoma $KDE_{scotoma}$ with the averaged density distribution of the full-field reference dataset $KDE_{ref}$:

$$KDE_{comp}=\frac{KDE_{ref}-KDE_{scotoma}}{KDE_{ref}+KDE_{scotoma}}.$$

Voxels associated with visual field areas affected by an (artificial) scotoma lack stimulation and, therefore, the characteristic signal required for a successful fitting procedure. These voxels will not survive statistical thresholding and will lead to a surplus of pRF center density in the full-field condition in these visual areas, which will result in positive values of the scotoma estimation curve.

Areas outside of the scotoma will have comparable pRF center density in full-field and scotoma conditions. As the scotoma condition yields smaller numbers of pRF centers (as foveal pRF centers are absent), the normalized KDE yields higher values in peripheral nonscotoma areas, compared with the full-field condition. This results in negative values in the scotoma estimation curve. The scotoma border is classified using a value of 0.1, as used in our previous implementation of the KDE approach (Hummer et al., 2018).

## Results

**Figure 1.**
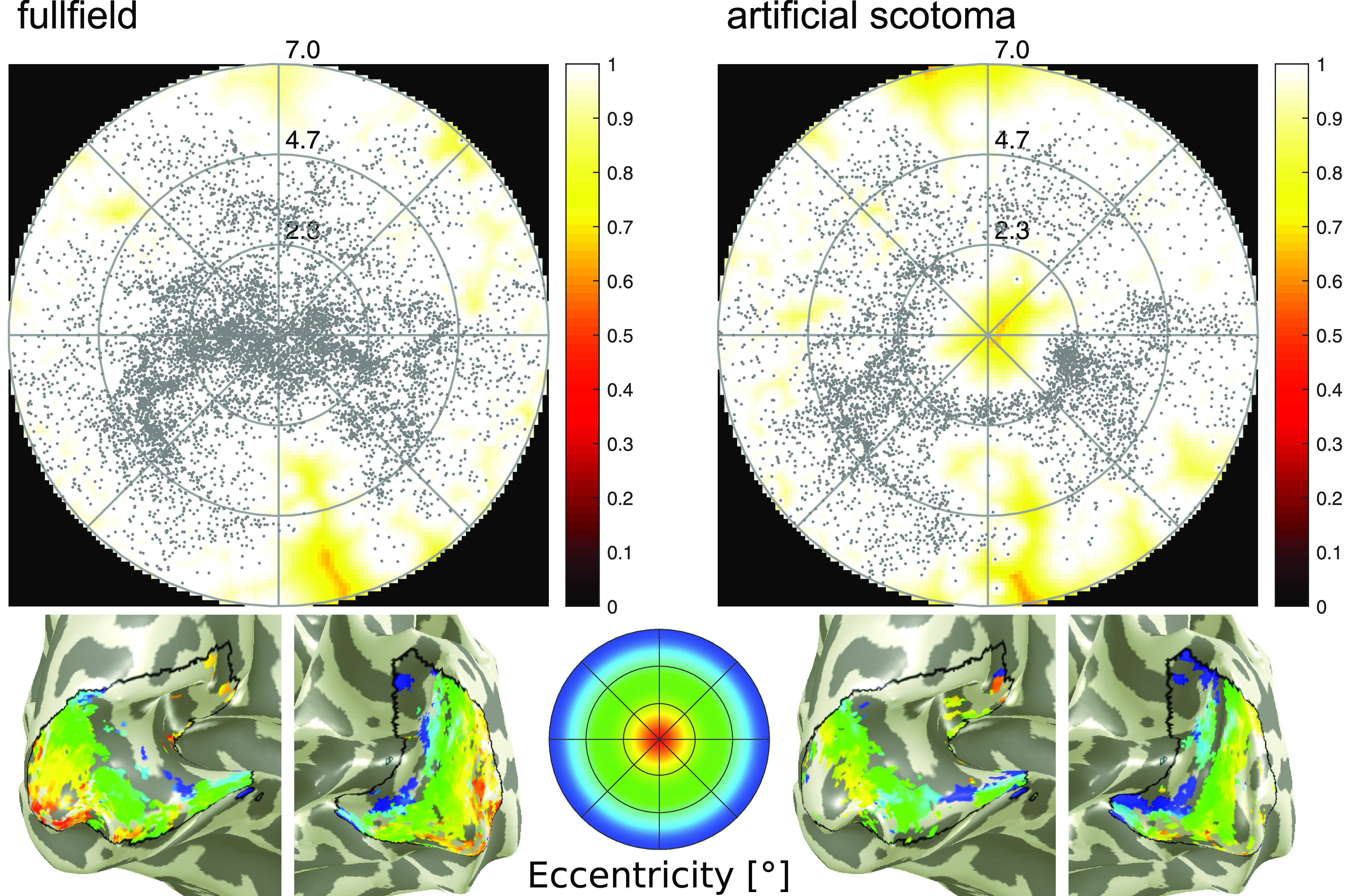
Results of the one subject where full-field (left) and artificial scotoma runs (right) were acquired. The top row shows coverage plots where each dot corresponds to a detected pRF center, while the bottom row shows eccentricity values overlaid to the visual cortex surface. The circles shown in the eccentricity color wheel match the circles of the coverage plots (2.3°, 4.7°, 7.0°).

A total of five subjects exceeded exclusion criteria thresholds and, therefore, the following reported results include a population of 15 subjects. Individual run results yielded the expected retinotopic organization, i.e., foveal visual fields are represented on the posterior part of the visual cortex while more peripheral areas are represented in more anterior regions. In all 60 respective single-run results (four runs per subject), the central artificial scotoma is clearly visible. Figure 1 shows a comparison between pRF results of full-field and artificial scotoma stimulation. While the pRF center density is highest in foveal areas and decreases in more peripheral regions with the full field stimulation, the artificial scotoma condition shows no pRF centers in the central visual field of the coverage map. Around the scotoma border, pRF center density is strongly increased. Additionally, the eccentricity parameter overlayed on the V1 in Figure 1, bottom row, shows no activation around the occipital pole corresponding to the foveal visual field (red and orange colors) in the scotoma condition, compared with coverage in the no-scotoma condition.

**Figure 2.**
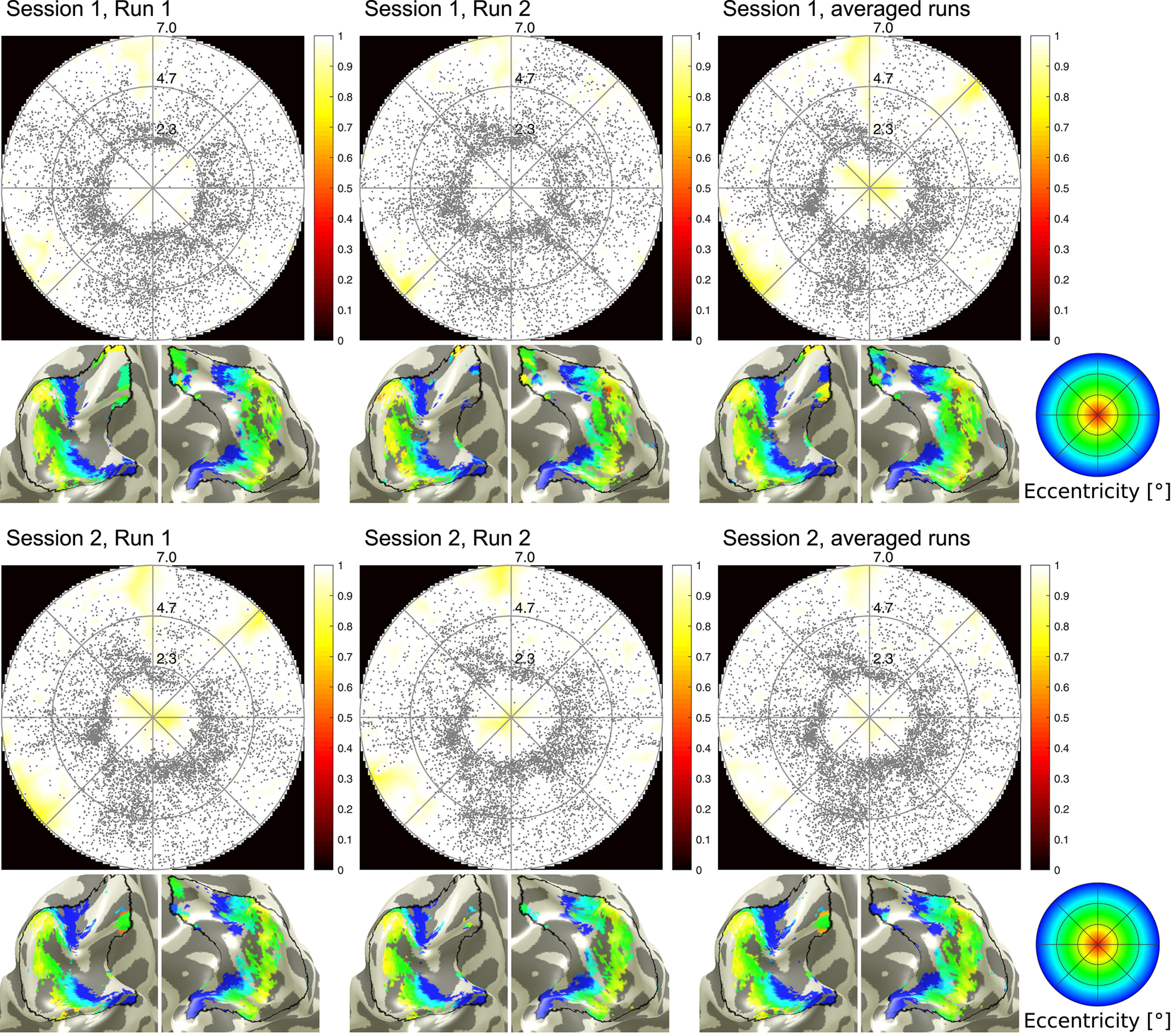
Comparison of scotoma mapping results (coverage maps and eccentricity maps overlaid to the cortical surface) across runs and sessions in a typical subject. Blank central areas in the coverage correspond to the foveal artificial scotoma (2° radius).

Results for an exemplary subject for the four respective runs are shown in Figure 2. Despite subtle differences in pRF center numbers within the central (scotoma) visual field, all maps clearly show the artificial scotoma in the respective coverage maps.

### Reproducibility

**Figure 3.**
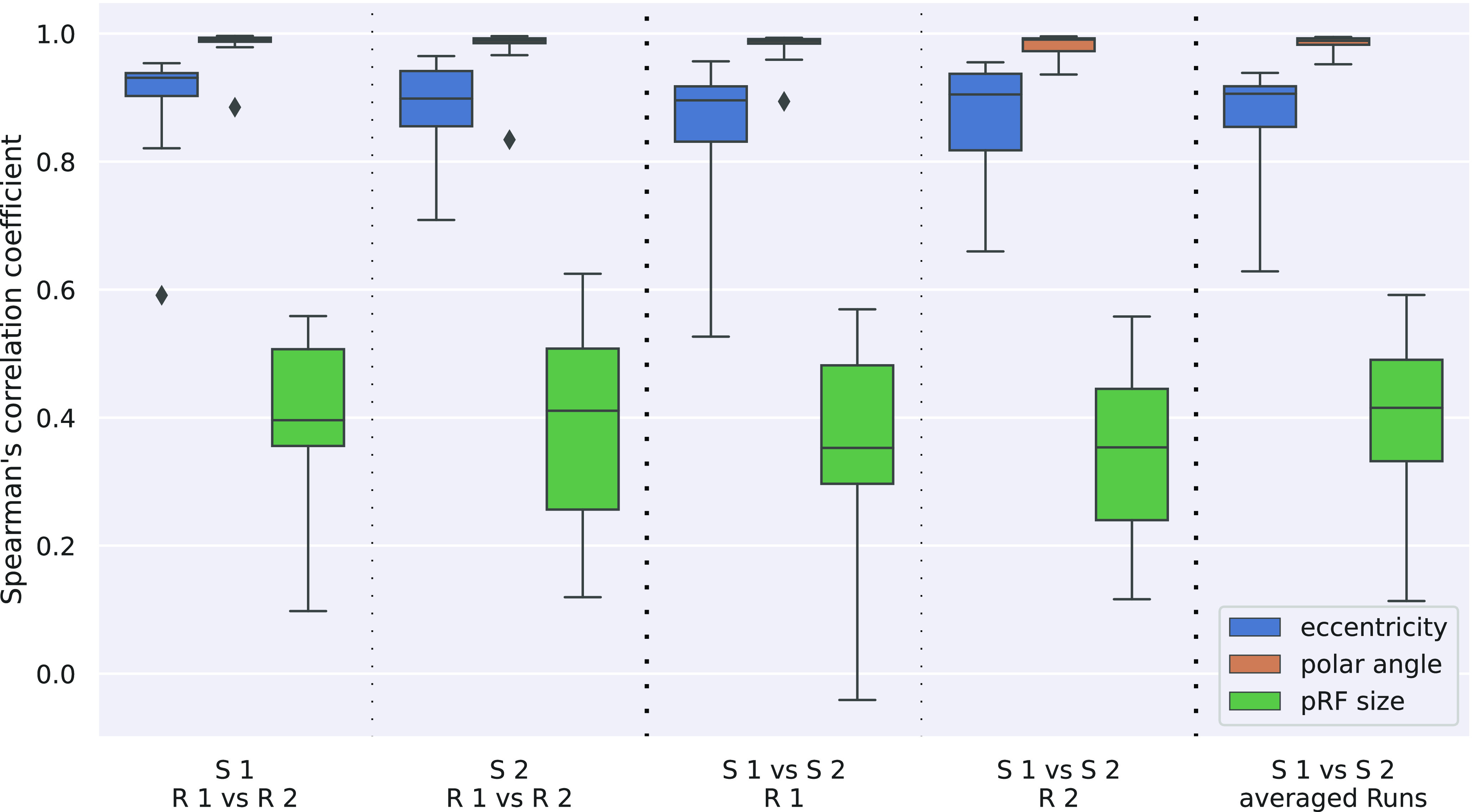
Spearman’s correlation coefficient for pRF center position (eccentricity and polar angle) as well as pRF size. Different columns correspond to the different run comparisons (columns 1 and 2, intrasession; columns 3–5, intersession).

**Figure 4.**
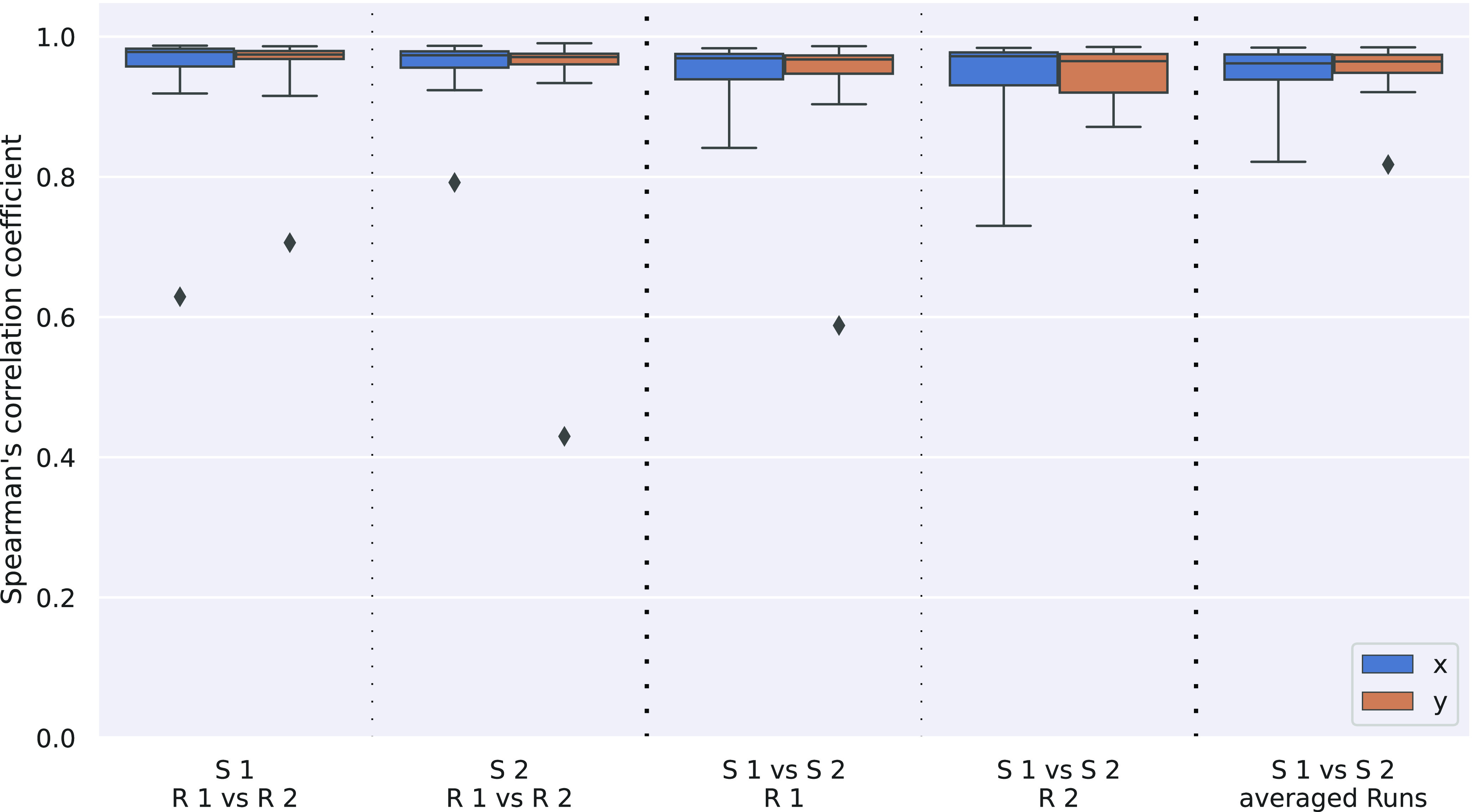
Reproducibility of the original Cartesian fitting parameters *x* and *y*. While correlation coefficients vary between eccentricity and polar angle parameters, they are remarkably similar for Cartesian parameters, for both intrasession and intersession comparisons.

Reproducibility was assessed by calculating Spearman’s correlation coefficient for the various combinations of runs and the results are shown in Figures 3 and 4. Intrasession reproducibility was assessed separately for the first and second session. Intersession reproducibility was calculated separately for the first and second runs of each session, as well as for the run-averaged session results.

10.1523/ENEURO.0087-22.2022.f3-1 Extended Data Figure 3-1 Spearman’s correlation coefficient for pRF center position (eccentricity and polar angle) as well as pRF size. Different columns correspond to the different run comparisons (columns 1 and 2, intrasession; columns 3–5, intersession). Data shown here originates from the five excluded subjects. Download Figure 3-1, EPS file.

Figure 3 shows correlation coefficients for eccentricity, polar angle and pRF size σ across the different comparisons performed. Averaged correlation coefficients are given in Table 1. It can be seen that correlations are highest for polar angles (all >0.98) and slightly lower for eccentricities (0.87–0.92). Correlations for σ (pRF sizes) are considerably lower (0.35–0.41), less than half compared with polar angle and eccentricity results. Also, the variance across subjects is higher for the pRF size parameter.

**Table 1:** Mean Spearman’s correlation coefficients averaged across all 15 subjects for the different intrasession and intersession conditions and pRF parameters

Correlation coefficients	Session 1 run 1 vs run 2	Session 2 run 1 vs run 2	Run 1 session 1 vs session 2	Run 2 session 1 vs session 2	Combined session 1 vs session 2
Eccentricity	0.916	0.903	0.879	0.890	0.886
Polar angle	0.989	0.988	0.986	0.986	0.988
pRF size	0.411	0.399	0.353	0.351	0.405
*x*	0.970	0.967	0.960	0.957	0.958
*y*	0.969	0.966	0.958	0.959	0.961

Simulation results, verifying the systematic shift of correlation values when switching from cartesian to polar coordinates are shown in Extended Data Table 1-1.

10.1523/ENEURO.0087-22.2022.tab1-1 Extended Data Table 1-1 Spearman’s correlation coefficients resulting from 1000 repetitions of pRF center position on a Cartesian grid for the full-field and scotoma condition. It can be seen that correlation values for radius are lower than polar angle, while Cartesian variables result in similar correlation values. The presence of a central scotoma increased the differences between the two coordinate systems. Download Table 1-1, DOCX file.

It is important to note that eccentricity and polar angle parameters are not explicitly estimated in the pRF analysis approach; rather they are calculated from the Cartesian *x*- and *y*-parameters obtained in the actual fitting process. Thus, correlations were also calculated for these Cartesian parameters and are plotted in Figure 4, with average values in Table 1. Here, the main observations are the very high correlation (0.95–0.97) across all comparisons and the remarkable similarity of *x*- and *y*-parameter correlation results. The systematic influence of the transformation between Cartesian and polar coordinates on the reproducibility values was confirmed by simulations and the results are reported in Extended Data Table 1-1.

For every parameter and combination of two runs, we calculated the across-subject mean difference over all voxels within the mask, either for all voxels (Table 2) or limited to voxels with eccentricity values <7° visual angle, i.e., within the stimulated field of view (Table 3).

**Table 2:** Mean differences within one voxel per parameter averaged across all 15 subjects for the different intrasession and intersession conditions and pRF parameters

Differences (SEM; °)	Session 1 run 1 vs run 2	Session 2 run 1 vs run 2	Run 1 session 1 vs session 2	Run 2 session 1 vs session 2	Combined session 1 vs session 2
Eccentricity	0.89 (0.08)	1.02 (0.10)	1.08 (0.09)	1.05 (0.11)	0.98 (0.08)
Polar angle	9.26 (1.48)	9.89 (1.46)	10.86 (1.41)	10.93 (1.13)	9.83 (0.85)
pRF size	0.53 (0.04)	0.58 (0.06)	0.61 (0.06)	0.57 (0.05)	0.54 (0.04)
*x*	0.77 (0.08)	0.90 (0.10)	0.92 (0.07)	0.94 (0.10)	0.83 (0.05)
*y*	0.76 (0.08)	0.84 (0.09)	0.93 (0.08)	0.88 (0.09)	0.84 (0.08)

All values in degrees visual angle. Values in parentheses show the SEM across subjects.

**Table 3:** Similar to **Table 2 but limited to voxels with eccentricity values below 7° visual angle, i.e., within the area stimulated**

Differences (SEM; °)	Session 1 run 1 vs run 2	Session 2 run 1 vs run 2	Run 1 session 1 vs session 2	Run 2 session 1 vs session 2	Combined session 1 vs session 2
Eccentricity	0.51 (0.04)	0.57 (0.05)	0.63 (0.04)	0.63 (0.06)	0.57 (0.04)
Polar angle	9.33 (1.07)	10.13 (1.27)	11.48 (1.36)	11.11 (1.01)	9.92 (0.75)
pRF size	0.46 (0.04)	0.48 (0.04)	0.53 (0.06)	0.48 (0.04)	0.46 (0.03)
*x*	0.50 (0.04)	0.58 (0.05)	0.63 (0.05)	0.64 (0.06)	0.55 (0.03)
*y*	0.49 (0.04)	0.53 (0.05)	0.62 (0.05)	0.59 (0.06)	0.55 (0.04)

All values in degrees (°). Values in parentheses show the SEM across subjects.

Reproducibility was also assessed for the five excluded subjects, as the exclusion criteria (lack of attention or unstable fixation and movement) could be regularly exceeded when applying this method to patients suffering from visual field loss. The results of the intersession and intrasession correlation coefficients are shown in Extended Data Figure 3-1. Reproducibility values for the different run comparisons overall yield lower but comparable values for the excluded subjects (eccentricity ∼0.73; polar angle ∼0.93; size ∼0.26).

**Figure 5.**
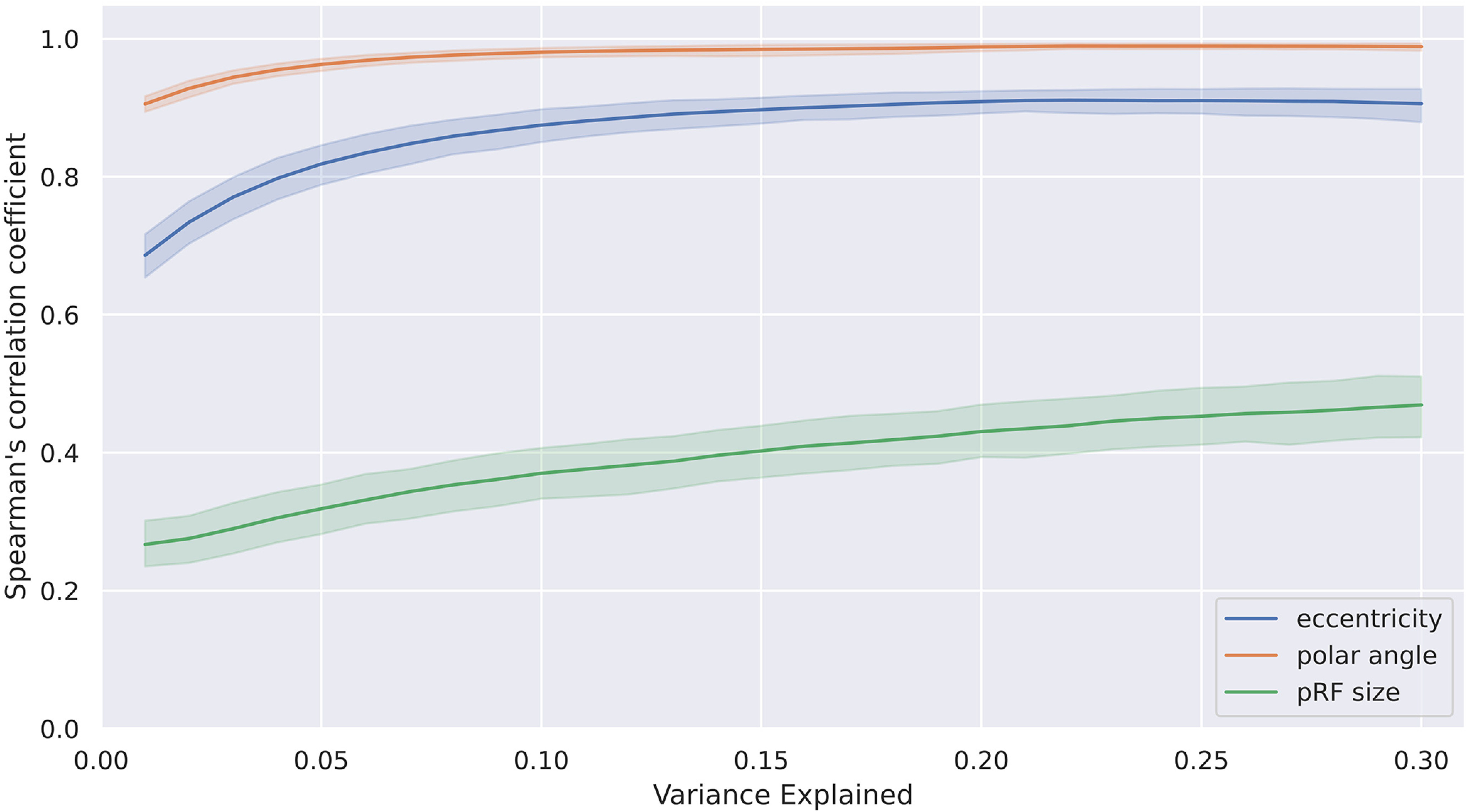
Change of reproducibility across voxel inclusion thresholds (variance explained). The plot shows Spearman’s correlation coefficients averaged across all single-run comparisons (intersession and intrasession conditions) with 95% confidence interval for the three pRF mapping parameters eccentricity, polar angle and pRF size.

An analysis regarding the influence of the chosen variance explained threshold (voxel inclusion criteria) on reproducibility was performed calculating the reproducibility using a range of different thresholds. Results are reported in Figure 5. It can be seen, that for thresholds below 10% the reproducibility for the pRF center position is quickly dropping while staying relatively stable above this value. The pRF size parameter is steadily increasing its reproducibility with variance explained threshold.

### Comparison of pRF modeling approaches

**Figure 6.**
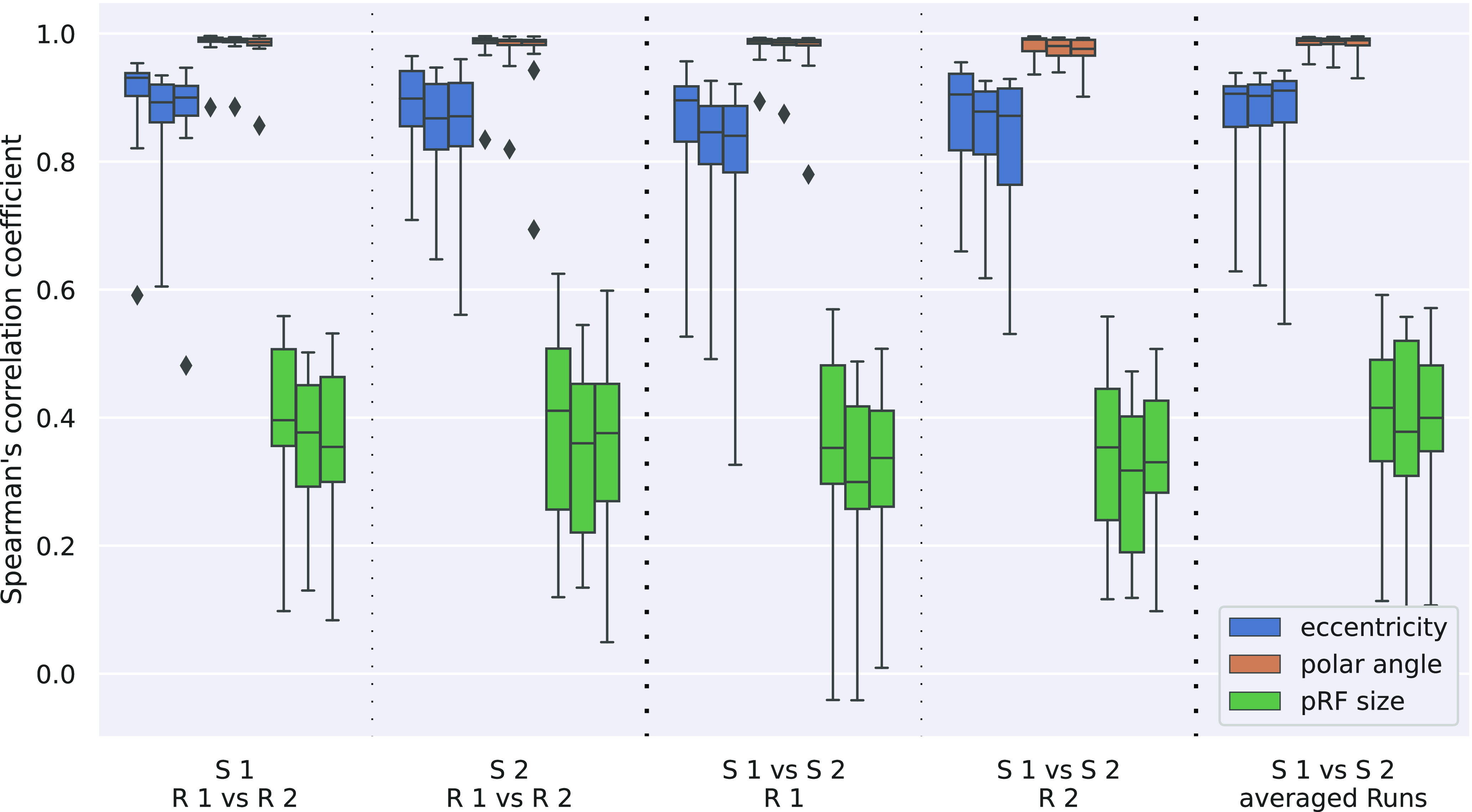
Comparison between the standard Gaussian model, the Gaussian model without HRF fit and nonlinear model fitting (left, middle, right results in each category). The values shown are Spearman’s correlation coefficient for all run comparisons and the three mapping parameters eccentricity, polar angle and pRF size.

Equivalent to the above, Spearman’s correlation coefficients were additionally calculated for pRF results obtained by the two comparison models. Figure 6 shows the reproducibility for the center position (eccentricity, polar angle) and pRF size. The three columns within every parameter show results for (1) standard Gaussian model, (2) standard Gaussian model with canonical HRF (i.e., without HRF fitting), and (3) nonlinear fit model, respectively. The two alternative models yielded lower correlation values and/or increased intersubject variance for the single-run comparisons for all parameters.

### Scotoma size estimation

**Figure 7.**
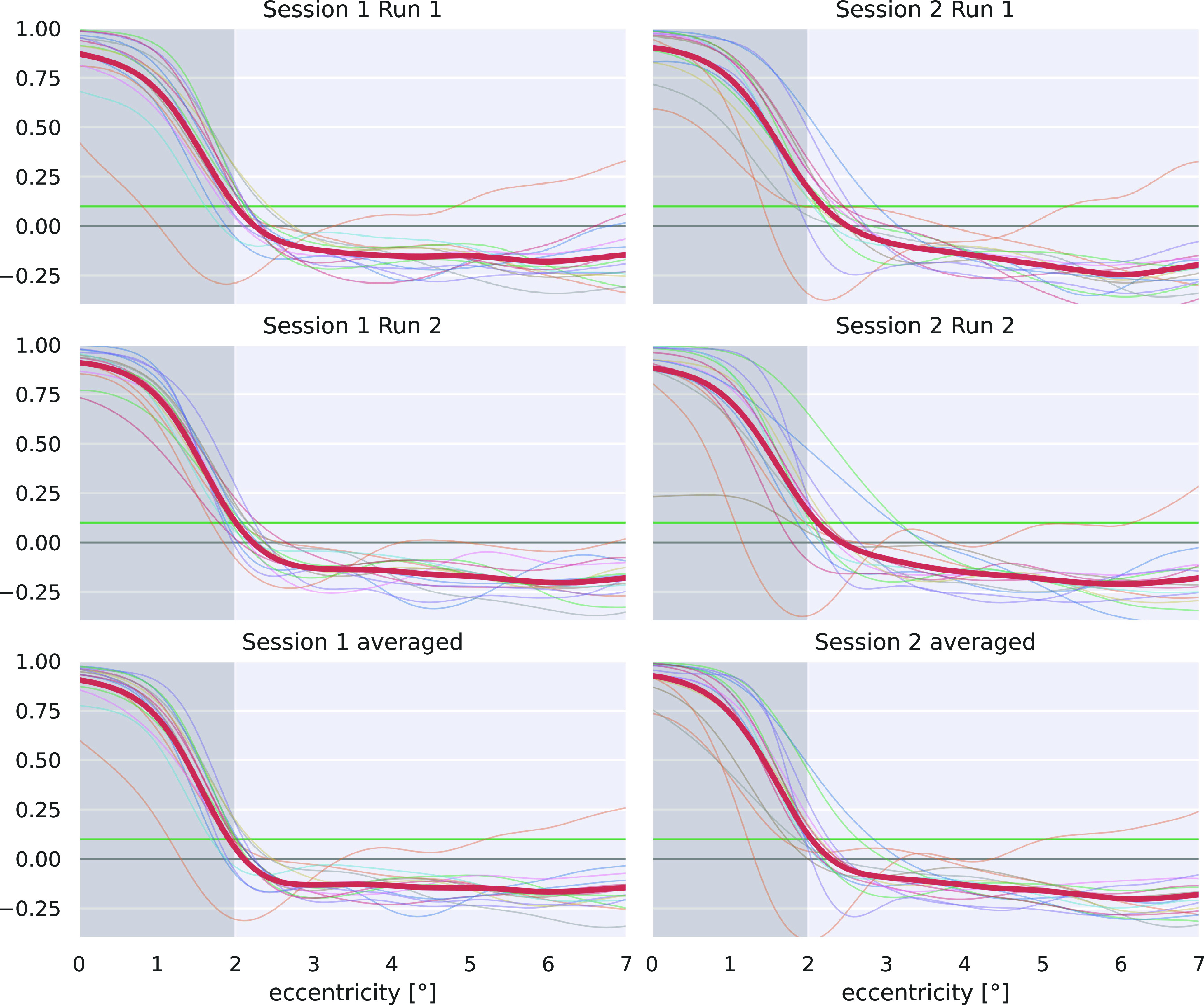
Scotoma estimation curves calculated as the relative difference of pRF center density between scotoma and reference stimulation. Every subplot shows results for all subjects (thin lines) and mean across subjects (bold red line) for single run or averaged analyses. The scotoma is indicated by the dark gray area below 2° radius. Values below zero (horizontal gray line) indicate a relative surplus of pRF centers in the scotoma condition, while values above indicate higher pRF center density in the reference data set. The scotoma detection border at 0.1 is indicated by a horizontal green line.

**Figure 8.**
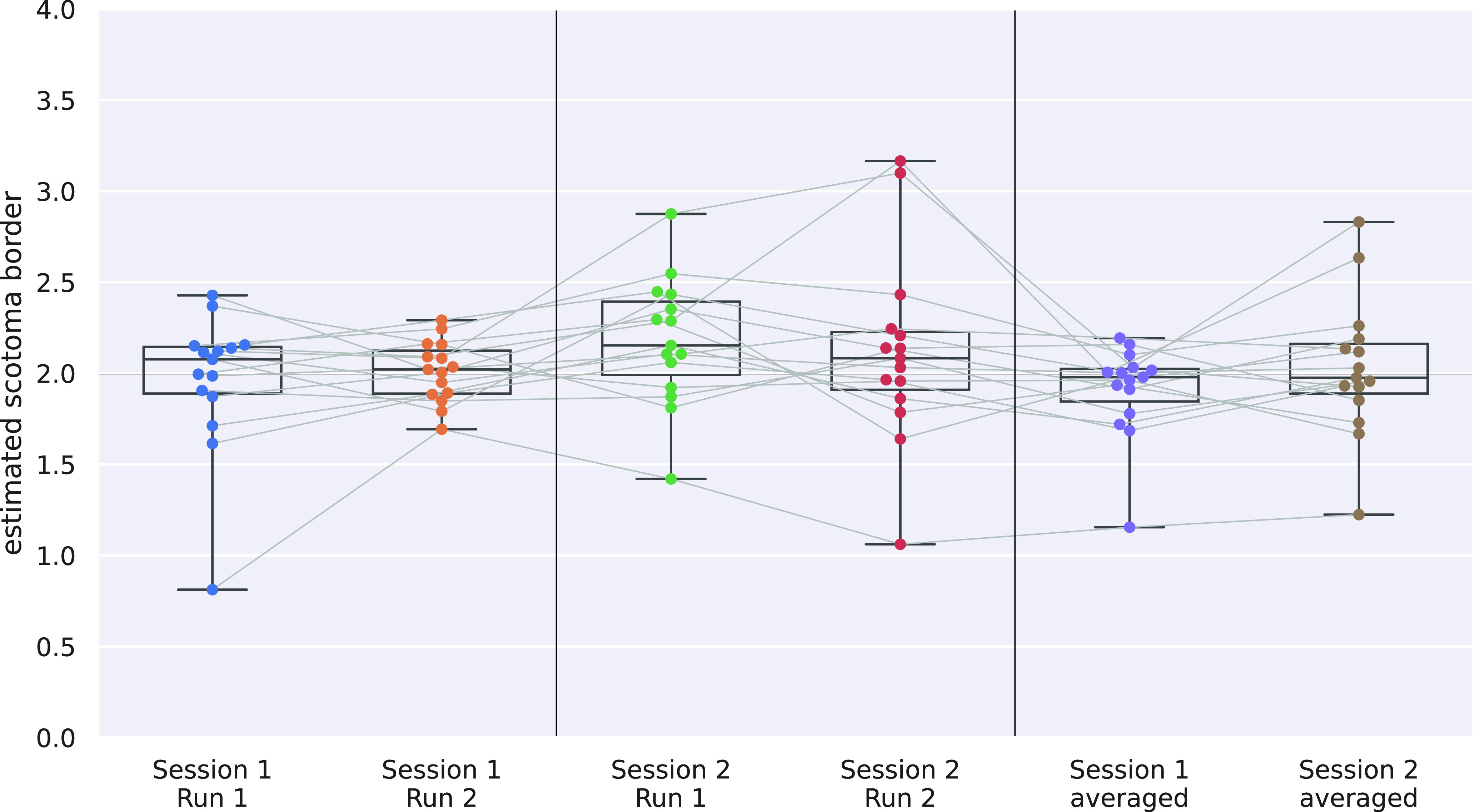
Estimated scotoma border values for the different single-run or average results. The horizontal thin gray lines connect the same subject’s results throughout all session results. Extended Data Figure 8-1 shows results for the other two tested pRF models.

Scotoma estimation lines for single subjects, as well as across-subject averages, are shown in Figure 7. The simulated scotoma with a radius of 2° is marked by the dark gray area. The radius, where the scotoma estimation line hits the value 0.1 (green line) marks the border of the central scotoma, as proposed by Hummer et al. (2018). All estimated scotoma border radii are shown in Figure 8 as distribution across subjects for single-run results. The distribution of the estimated borders across all single-subject results is not significantly different from the artificial scotoma size of 2° (one-sample *t* test: *p* = 0.338). Additionally, scotoma size estimation results for the two pRF models (canonical HRF, nonlinear fitting) yielded similar results and are reported in Extended Data Figure 8-1.

10.1523/ENEURO.0087-22.2022.f8-1 Extended Data Figure 8-1 Estimated scotoma border for all subjects and measured runs. In contrast to Figure 8, where scotoma borders estimated from the standard Gaussian modelling are reported, the comparison models are shown: (***A***) Gaussian model without HRF fit; (***B***) fit considering spatial nonlinearities. Download Figure 8-1, EPS file.

Scotoma border estimations from the averaged across subjects’ classification as well as the mean difference from the ground-truth scotoma size are reported in Table 4, separated for the four single-run and two averaged results. Subject-averaged scotoma estimations are very close to the ground-truth value of 2° radius with a maximum difference of ∼0.2°. Results obtained in the first session yield closer scotoma estimations as well as a smaller spread across the group compared with session two results. Averaged results for the scotoma border result in smaller estimated scotoma values, as well as a reduced mean deviation and therefore more uniform results for the second session.

**Table 4:** Scotoma borders estimated from averaged across subjects classification curves for all runs

Analysis	Average scotoma border (° radius)	Mean difference (° radius)
Session 1, run 1	2.01	0.25
Session 1, run 2	2.00	0.12
Session 2, run 1	2.19	0.31
Session 2, run 2	2.10	0.36
Session 1, averaged	1.92	0.16
Session 2, averaged	2.04	0.26

The second column reports the mean difference from the ground truth scotoma border across subjects

## Discussion

In this study, we assessed the intrasession and intersession reproducibility of pRF-mapping derived artificial scotoma estimates. In two separate sessions, young healthy participants maintained central fixation while being shown a moving bar stimulus pattern revealing flickering checkerboards.

The artificial scotoma was simulated by masking the central 2° radius of the visual field, mimicking visual field losses in the central areas as observed in various patient groups suffering from retinal disease. This artificial scotoma approach provides the ground-truth conditions required for assessing detection accuracy.

Coverage maps obtained from artificial scotoma runs show increased pRF center density at the scotoma border. This pile-up is a result of the modeling approach intrinsic to pRF analysis when full-field stimulation is assumed. It will force a shift of pRF centers from inside the scotoma toward the nearest border as demonstrated by Binda et al. (2013). Following this, some pRF centers from inside the scotoma will appear at the scotoma border. The same behavior is also expected in patients with real scotomata. To compensate for these shifting effects, the scotoma could be included within the analysis, which would allow for fitting voxels at their correct position within the scotoma border. However, this is not expedient in our case as we would like to assess the performance of pRF mapping techniques to map unknown scotomata.

While polar angle and eccentricity yielded very high reproducibility values (>0.98 and >0.87, respectively), reproducibility in pRF size showed much lower values (∼0.40). These lower correlations in the size parameters could be caused by a number of different reasons. It has been shown recently that the size fit is heavily influenced by the subject-specific HRF fitting routine (Lerma-Usabiaga et al., 2020), which was performed independently for each run with the standard model approach. We took a closer look at this issue and found that the difference of estimated HRFs between corresponding runs yields only small differences (first peak position ±0.8 s; width of first peak ∼1 s difference). If indeed poor HRF fits would reduce reproducibility in pRF sizes, analyses with canonical HRF models should be beneficial. However, our results show that this is not the case as correlations are slightly reduced when mrVista’s HRF fitting procedure is not included in the analysis (Fig. 6). This indicates a small but beneficial impact of individual HRF fitting procedures. Improved HRF estimations might be possible with additional measurements dedicated to this task or simultaneous pRF and HRF estimations (for review, see Lindquist et al., 2009). Another option for possible improvements in HRF and pRF size fitting quality could be the use of randomized, rather than predictable stimulus apertures. This, however, is associated with a loss in BOLD signal-to-noise ratio and subsequently needs longer stimulation times (Lerma-Usabiaga et al., 2020).

Comparing these results with previous assessments of the pRF method’s reproducibility by van Dijk et al. (2016) where intersession Spearman correlation was calculated for averaged runs (eccentricity: 0.90; polar angle: 0.83; pRF size: 0.36), we find almost similar results (eccentricity: 0.886; polar angle: 0.988; pRF size: 0.405). This similarity in reproducibility results is remarkable given the considerable difference in fMRI acquisition parameters. Here, we used a 7T MRI scanner with an isotropic voxel size of 1 mm, compared with 1.5T with 2.3 mm voxels in the van Dijk study, resulting in a factor >4 in magnetic field strength and a factor of 12 in voxel size. Further, the stimulation paradigm differed as we used a moving bar compared with the combined wedges and rings paradigm in their experiment (Alvarez et al., 2015), which also impacts pRF results (Linhardt et al., 2021). Despite all the differences, the reproducibility results are highly similar for the eccentricity and size parameter estimates.

With respect to polar angle reproducibility, our results yield higher values compared with previous studies. This, however, can be explained using artificial scotomata in this study as the effect of a shift of the pRF center position depends on the associated eccentricity. A central voxel shifted by, e.g., 0.5° visual angle can change its polar angle by up to 180° while the same shift in more peripheral visual field has little influence on the resulting parameter regions (0.5° shift at 7° radius results in only 4° polar angle change). Since the use of a central artificial scotoma in this study, macular areas are underrepresented, polar angle reproducibility will be increased. Reproducibility values are almost equal for *x* and *y* (>0.95). Besides the fact that the coordinate system transformation introduces a systematic bias of reproducibility values, this effect was found to be increased through the introduction of a central scotoma. Both effects were confirmed using simulation and the results are reported in Extended Data Table 1-1. Following these biases, a reliability assessment using *x* and *y*-parameters as pRF center position seems most appropriate, as it also represents the original fitting space.

Taken together, our results corroborate previous pRF reproducibility studies in suggesting that the pRF size parameter is by far the least reproducible parameter in pRF mapping, leaving space for future improvements in the field. Therefore, interpretation of pRF data should rather be driven by the pRF center position than the size.

Since the herein used inclusion criteria were quite strict and might not be applicable in clinical studies, we also assessed reproducibility in the subjects that were excluded because of movement and/or insufficient fixation performance/attention as a proxy for patient studies. Extended Data Figure 3-1 shows the average reproducibility in the excluded group. While correlation values are lower compared with the actual study population, eccentricity (∼0.73 vs >0.87) and polar angle results (∼0.93 vs >0.98) are quite comparable. Still, pRF size reproducibility is poor (∼0.26 vs ∼0.40).

For the main analysis, we used a standard symmetric 2D Gaussian function as pRF model, which was advantageous compared with asymmetric Gaussian functions for early visual areas (Lerma-Usabiaga et al., 2021). However, some studies have found higher variance-explained values, and therefore fitting quality when incorporating surround suppression in the pRF shape (Zuiderbaan et al., 2012), although no connection between the surround suppression on a neuronal level (Hubel and Wiesel, 1965) and a voxel-level has been found yet. Further, introducing a parameter encoding for spatial nonlinearity in the fitting procedure might be an option to prevent pRF size overestimation (Kay et al., 2013). We tested for effects of nonlinear fitting on V1 pRF results and found that neither reproducibility nor scotoma size estimation benefitted from this approach. Since surround suppression effects are considered stronger in early visual areas, the implementation of novel pRF models, including both effects, as Aqil et al. (2021), could improve reproducibility for pRF parameters or scotoma estimation even further.

Although the artificial central scotoma cannot always clearly be seen from the visual field coverage (color coded in the coverage map) alone, the scotoma can be delineated from the accumulations of pRF centers around the scotoma border, compared with a more uniform center density in full-field condition. Simulations in Binda et al. (2013) showed the effects on pRF centers when scotoma is introduced in the measurement, but not modelled in the analysis: central pRF sizes tend to increase, while noncentral pRF centers are shifted to more eccentric positions. These effects, together with missing stimulation inside the scotoma, explain the missing presence of above-threshold voxels within the scotoma and the increased center density at the scotoma border. To compensate for these shifting effects, the scotoma could be included in the analysis, which would allow for fitting voxels on their correct position within the scotoma border. However, this is not expedient in our case, since future studies on patients suffering from retinal disease cannot incorporate this step because of the nonexistent knowledge of the scotoma ground truth.

We make use of the described effects of center shifting toward outside of the scotoma and the missing above-threshold pRFs for the classification of the scotoma border as previously described by Hummer et al. (2018). Although in this study we used a different, independent dataset as a reference, compared with the original study (Hummer et al., 2018) where scotoma and full-field data were obtained in the same subjects, we found remarkably accurate scotoma estimations on a group level. This allows for future studies for the estimation of scotoma on data, where the ground truth is not known. In patients suffering from retinal diseases, no full-field stimulation can be acquired, but scotoma extent could be estimated by comparisons to the reference datasets.

Although the signal-to-noise ratio is increased by averaging runs within sessions, it yielded only marginal improvements in correlation values. Further, there is no clear benefit in scotoma size estimation, and it may be questioned whether the observed benefits justify the doubling in measurement time needed for averaging two runs. Correlation values for the intersession comparison, as well as the spread between subjects in the scotoma size yield slightly lower results, compared with the intrasession or first session results. This could be explained by the preprocessing layout, where the second session goes through an additional realignment step. Still, it may be concluded that pRF mapping results are highly stable, regardless of head positioning or time between sessions. This is a very important finding regarding future goals to use the method for longitudinal studies in patients suffering from retinal disease or when combining mapping results acquired in different sessions.

To assess the effects of variance explained threshold selection, we repeated all correlation calculations for thresholds from 1% to 30%. The results are shown in Figure 5. For thresholds above 10% the reproducibility of the pRF center position stays almost constant. Below 10% variance explained threshold, mean correlation values are reduced. For pRF sizes, correlation steadily increases with higher thresholds.

Additional analyses, e.g., the dependency of reproducibility in different eccentricity bins of the visual field of view were not included in this work, since recent findings (Stoll et al., 2022) suggest systematic influences of the binning method when multiple runs are included. In this case, the voxels for the computation of correlations within an eccentricity band would be chosen based on one run, with the other run not considered, biasing the results. To a smaller extent, this is also the case when applying variance explained thresholds to one subject before the correlation calculation. This limitation was minimized by only including voxels exceeding thresholds for both runs.

Subjects were instructed to strictly focus on the central fixation dot and were filtered for insufficient fulfilment of the fixation task. However, some runs yield central activations, which could be caused by unstable fixation. A major limitation of the described study is the missing fixation tracking by an eye-tracking and gaze stability correction system, as implemented by Hummer et al. (2016). Future studies will heavily benefit from the implementation of standardized online eye-tracking on 7T MRI scanners.

In conclusion, our results clearly show that mapping pRF centers in artificial scotoma studies yield highly reproducible results. The width of pRF as estimated by the pRF size is less reproducible. Further, we conclude that automated estimation of artificial scotoma sizes is possible in subjects where no full-field stimulation data are available by using separate reference pRF datasets.

## Synthesis

Reviewing Editor: Nicholas J. Priebe, University of Texas at Austin Decisions are customarily a result of the Reviewing Editor and the peer reviewers coming together and discussing their recommendations until a consensus is reached. When revisions are invited, a fact-based synthesis statement explaining their decision and outlining what is needed to prepare a revision will be listed below. The following reviewer(s) agreed to reveal their identity: Brian Wandell, David Ress.

Synopsis

Two reviewers evaluated the manuscript “Intra- and intersession reproducibility of artificial scotoma pRF mapping results at ultra-high fields” and both reviewers agreed that the manuscript provides a solid contribution to the field and is well-written. The authors test the reproducibility of their mapping artificial central “scotoma” using previously developed pRF methods, and demonstrate excellent accuracy and reliability. The reviewers have made minor suggestions, listed below, to improve the overall clarity of the manuscript.

Comments:

1) In “MRI measurements", it would be helpful to know the field-of-view and the echo spacing in order to calculate the total EPI acquisition time. The data given suggest that the acquisition time was quite long, ∼50 ms, which is substantially longer than T2* at 7T, and therefore likely to introduce significant T2* blurring/distortion. If so, some justification or acknowledgement of this issue is needed.

2) The conclusion states: “Further, we conclude that automated size estimation of artificial scotomas is also possible in subjects where no fullfield stimulation reference data is available.” This is probably true from inspecting Figures 1 and 2. But given that most of the analyses use the full field and the scotoma (Equation on Line 238), it is not clear which analysis is being referred to for this claim.

3) The scotoma border estimation procedure is described mathematically, but could use a paragraph that outlines the logic involved in the procedure, particularly the apparently arbitrary choice of 0.1 as a border criterion for KDE.

4) Both reviewers noted the data presentation in figs 1 and 2 could be improved. The dashed black line in the color bar for eccentricity in Figs. 1 and 2 are not adequately visible given the size. Also, perhaps you could put in a series of circles, rather than defining eccentricity by the numbers at the bottom. The color overlay is not clearly described. It should either be removed or the text expanded to make use of it.

5) The “pile up” of representation at the artificial scotoma border is an interesting phenomenon that seems worthy of further discussion. Why might this occur, and how might this feature observed in healthy subjects carry over to those with real scotomas?

6) This work was carried out with a symmetric central artificial scotoma, whereas real scotomas could be peripheral and asymmetric. How might such realistic scotomas affect the stability of the measurement? Moreover, how effectively will this artificial scotoma characterization carry over to realistic measurements of pathology?

7) It would be useful to know more about the excluded subject data. For applications to patients, such failures might be common. One minor suggestion is to describe the data for those individuals, perhaps in a supplementary section.

8) Line 212: It might be better to include at least one figure - or at least a comment - that shows what happens to your main measures if you vary the pixel-inclusion criterion (10% explained).

9) Line 218: where -> were

10) Line 397: It might be useful to put in a trivial simulation to point out how noise in Cartesian translates into noise in angular. A supplementary figure, or at least some quantification on your own, might be helpful. The code would be something like this:

[X1,Y1] = meshgrid(-7:1:7,-7:1:7); [T1,R1] = cart2pol(X1,Y1);

% Add noise

X2 = X1 + randn(size(X1)) * 0.5; Y2 = Y1 + randn(size(Y1)) * 0.5;

% Convert to polar in radiances (theta, rho)

[T2,R2] = cart2pol(X2,Y2);

figure; subplot(2,2,1); plot(X1,Y1,’ko’,X2,Y2,’r.’); grid on; xlabel(‘deg’); ylabel(‘deg’);

% Position

subplot(2,2,2); scatter(X1(:),X2(:)); grid on; title(‘Position’); xlabel(‘deg’); ylabel(‘deg’);

% Eccentricity

subplot(2,2,3); scatter(R1(:),R2(:));grid on; title(‘Eccentricity’); xlabel(‘deg’); ylabel(‘deg’);

% Angular

% Get rid of the edges at -pi and pi

l1 = (T1 < (pi - 0.1)) & (T1 > -pi + 0.1);

l2 = (T2 < (pi - 0.1)) & (T2 > -pi + 0.1);

l = l1 & l2;

T1d = rad2deg(T1(l));

T2d = rad2deg(T2(l));

subplot(2,2,4);scatter(T1d(:),T2d(:));grid on; title(‘Angular’); xlabel(‘angular deg’); ylabel(‘angular deg’)
