# Supplementary material for: Intrasession and Intersession Reproducibility of Artificial Scotoma pRF Mapping Results at Ultra-High Fields
Source: eNeuro. 2022 Sep 21;9(5):ENEURO.0087-22.2022. doi: 10.1523/ENEURO.0087-22.2022 (PMC9512620; doi:10.1523/ENEURO.0087-22.2022)
Supplement: Extended Data Table 1-1 — Spearman’s correlation coefficients resulting from 1000 repetitions of pRF center position on a Cartesian grid for the full-field and scotoma condition. It can be seen that correlation values for radius are lower than polar angle, while Cartesian variables result in similar correlation values. The presence of a central scotoma increased the differences between the two coordinate systems. Download Table 1-1, DOCX file. [file enu-eN-CFN-0087-22-s04.docx]

|  | x | y | radius | polar angle |
| --- | --- | --- | --- | --- |
| No Scotoma | 0.9648 | 0.9648 | 0.8351 | 0.9825 |
| 2° Scotoma | 0.9665 | 0.9665 | 0.7933 | 0.9918 |
